# Management protocol for extremely/very low birth weight infants: impact of waterbed-style nesting combined with continuous breast milk feeding quality improvement on infants’ quality of life and neurobehavioral outcomes

**DOI:** 10.3389/fped.2026.1773947

**Published:** 2026-04-28

**Authors:** Bifei Zhang, Jufang Ding, Jing Chen

**Affiliations:** Neonatal Intensive Care Unit, Children’s Hospital of Soochow University, Suzhou, China

**Keywords:** breast milk feeding, continuous quality improvement, extremely/very low birth weight infants, neurobehavioral, quality of life, waterbed-style bird's nest

## Abstract

**Objective:**

To investigate the clinical efficacy of waterbed-style nests combined with breast milk feeding quality continuous improvement on early growth and neurobehavioral outcomes in extremely/very low birth weight infants, providing theoretical support for clinical practice.

**Methods:**

A retrospective study enrolled 103 extremely/very low birth weight infants admitted to our hospital from May 2024 to October 2025. Based on electronic medical records, they were divided into a control group (*n* = 50, conventional management) and an observation group (*n* = 53, water-bed nest combined with continuous improvement of breast milk feeding quality). Clinical outcomes during hospitalization were collected and compared: Short-term physical growth outcomes (weight gain rate, head circumference gain rate, length gain rate, time to regain birth weight, sleep duration), Neurobehavioral development [Neurobehavioral Assessment of the Neonate (NABA)], Serum indicators [total protein (TP), albumin (ALB), prealbumin (PA)], Complication occurrence (feeding intolerance, necrotizing enterocolitis, bronchopulmonary dysplasia, late-onset sepsis).

**Results:**

The observation group showed significantly higher weight gain, head circumference and length growth rates, and shorter time to regain birth weight (all *P* < 0.05). Neurobehavioral scores (primitive reflexes, muscle tone, general condition, functional capacity) and serum nutritional markers (TP, ALB, PA) were also higher post-intervention (*P* < 0.05). Incidences of feeding intolerance (20.75% vs. 46.00%) and bronchopulmonary dysplasia (5.66% vs. 20.00%) were significantly lower in the observation group (*P* < 0.05). No significant differences were found in necrotizing enterocolitis or late-onset sepsis.

**Conclusion:**

The waterbed-style bird's nest combined with continuous quality improvement in breast milk feeding effectively enhances feeding and nutritional status, promotes early physical growth and neurobehavioral function, and reduces short-term complications such as feeding intolerance and bronchopulmonary dysplasia in infants. Long-term follow-up studies are warranted to determine if these early benefits translate into sustained improvements in quality of life.

## 1 Introduction

Extremely preterm infants encompass very low birth weight (VLBW) and extremely low birth weight (ELBW) infants, defined as newborns weighing less than 1,500 g and 1,000 g respectively within the first hour of life. They represent the most vulnerable and challenging cohort in the neonatal intensive care unit (NICU). Their underdeveloped organ systems render them highly susceptible to complications such as necrotizing enterocolitis, retinopathy of prematurity, hearing impairment, and severe neurodevelopmental disorders following forced separation from the maternal uterus (1, 2). With rapid advances in perinatal medicine and neonatal intensive care techniques, alongside the establishment of maternity wards and early intervention programs in neonatology, survival rates for extremely low birth weight (ELBW) and very low birth weight (VLBW) infants have reached 50%–60%, with some hospitals reporting rates as high as 70% (3). However, survival is not the ultimate goal. Neglected care after discharge leads to growth retardation, intellectual disability, and adverse events in some extremely/very low birth weight infants, causing harm and burden to the children themselves and their families and society (4). Therefore, adopting scientifically effective methods to improve their quality of life and promote neurobehavioral development has become a global focus in neonatal medicine.

Previous studies indicate that among numerous environmental stressors in the NICU, gravitational forces causing postural discomfort, lack of boundary perception, and frequent painful procedures are primary stress sources. These can trigger significant fluctuations in vital signs, increased energy expenditure, and decreased blood oxygen saturation. Long-term accumulation may even adversely affect developing brain structures (5). Fang R et al. (6) noted that developmental supportive care emerged as a response to this challenge, centered on creating a comfortable, safe, womb-like environment for preterm infants by mimicking intrauterine conditions. Nest-style care, an internationally popular developmental approach for preterm infants, has gained traction in China in recent years as a safer new model of care. The waterbed nest represents an innovation and advancement of this approach. It not only provides tangible physical boundaries, imparting a sense of security through envelopment, but also simulates the maternal uterine environment through water temperature and gentle water wave oscillations, replicating the rhythmic vibrations generated by maternal movement during walking (7, 8). Vadakkan AJ et al. (9) indicate that this continuous, gentle rhythmic stimulation from waterbed nests is believed to positively stabilize premature infants’ physiological states, soothe their emotions, and promote the organization of sleep-wake cycles. This may provide an optimized environmental foundation for the orderly development of the nervous system. On the other hand, nutrition and feeding constitute another major pillar determining the quality of life for extremely low birth weight infants. Breast milk is the ideal food for newborns, and breast milk feeding is particularly crucial for preterm infants. It can reduce the incidence of feeding intolerance, late-onset sepsis, and necrotizing enterocolitis, prevent retinopathy of prematurity, and promote neurological development (10). However, a significant gap exists between ideal and reality. In clinical practice, breast milk feeding for extremely low birth weight infants faces challenges across the entire chain—from milk collection, storage, and transportation to fortification and feeding implementation (11). Therefore, merely advocating for breast milk feeding is insufficient; the focus must shift from whether breast milk feeding occurs to systematically improving the quality of the entire breast milk feeding process. Current domestic and international research predominantly focuses on evaluating the efficacy of single interventions or solely examines nutritional outcomes. Clinical studies investigating the application of waterbed-style nests combined with continuous quality improvement in breast milk feeding for extremely low birth weight infants are scarce.

Based on this, this study analyzes extremely low birth weight infants in our hospital. By examining the potential impact of waterbed-style nests combined with continuous quality improvement in breast milk feeding on these infants, we aim to provide theoretical guidance for clinical practice.

## 2 Materials and methods

### 2.1 Ethical statement

This study was approved by the institutional review board and ethics committee. Given its retrospective nature and use of de-identified data, informed consent was not required as it posed no risk or adverse impact on subjects’ treatment. This exemption complies with regulations and ethical guidelines pertaining to retrospective research.

### 2.2 Study design

This was a single-center, non-randomized quasi-experimental study with a retrospective before-after design. It included 103 extremely low birth weight (ELBW) and very low birth weight (VLBW) infants admitted to our institution. A practice change was implemented in our NICU in January 2025, transitioning from conventional management to a new protocol that included water-bed nests and continuous quality improvement in breast milk feeding. Consequently, participants admitted between May 2024 and December 2024 were assigned to the control group (*n* = 50, conventional management), and those admitted between January 2025 and October 2025 were assigned to the observation group (*n* = 53, new protocol). Group allocation was based on the date of admission relative to this practice change, as documented in the electronic medical record system.

### 2.3 Inclusion criteria

Inclusion criteria: (1) Meets the diagnostic criteria for extremely low birth weight infants outlined in the Consensus on Genetic Metabolic Disease Screening for Preterm, Low Birth Weight, and Ill Infants (12), with birth weight <1,500 g; (2) Gestational age between 28 and 36 weeks; (3) Hospital admission within 24 h of birth; (4) Postmenstrual age at the time of study enrollment >3 days, with stable vital signs; (5) All cases from singleton pregnancies.

Exclusion Criteria: (1) Concurrent congenital genetic metabolic disorders or congenital gastrointestinal malformations; (2) Concurrent severe functional impairment of cardiac, hepatic, renal, or other organs; (3) Hospitalization duration <14 days; (4) Maternal severe illness, infectious disease, or other clear medical contraindications to breast milk feeding; (5) Severe cardiac, hepatic, or renal dysfunction.

### 2.4 Methods

#### 2.4.1 Control group

Implement conventional management protocols: (1) Nutritional Support: Strictly adhere to relevant standards outlined in the Feeding Guidelines for Extremely Low Birth Weight Infants (13) throughout all stages. For breast milk feeding, mothers were encouraged to provide expressed breast milk, but no structured education, pumping schedule, or quality improvement protocols were implemented. Feeding was initiated based on physician orders, and breast milk, when available, was administered via nasogastric tube without standardized fortification or storage protocols beyond basic hospital guidelines. For infants exhibiting feeding intolerance, provide parenteral and enteral nutritional support based on recommendations from the Clinical Diagnosis and Treatment Guidelines for Feeding Intolerance in Preterm Infants (2020) (14), tailored to the infant's physical condition. (2) Thermal Management: Infants were first transferred to far-infrared radiant warmers for rewarming. Once body temperature reached ≥36 °C, they were moved to premature infant incubators. Clothing was removed to facilitate warmth retention and clinical observation. All procedures—including nutritional support and medication administration—were performed within the incubator. Infants were positioned to maintain a neutral thermal environment. Regular bathing was conducted, and relative humidity was maintained at 70%–75%. (3) Respiratory Care: After birth, thoroughly clear respiratory secretions. Oxygen therapy was administered to target peripheral oxygen saturation (SpO_2_) levels between 90% and 95%, in accordance with current neonatal resuscitation guidelines. The specific oxygen concentration was titrated to achieve this target range. Discontinue oxygen once the target SpO_2_ is consistently maintained in room air. Administer prompt medication for respiratory complications; implement mechanical ventilation for severe cases. (4) Infection Control: Nurses strictly adhere to aseptic technique during all procedures. Incubators, nasogastric tubes, humidifier bottles, and other equipment undergo regular disinfection. Saline solution is used to wipe the infant's palpebral fissures and oral cavity at scheduled intervals. Standard infection control practices for cord care and skin care were followed as per hospital protocol.

#### 2.4.2 Observation group

Building upon the control group, the observation group implemented water-bed-style nests combined with continuous improvement in breast milk feeding quality. (1) Water-bed-style nest: Adjusted incubator temperature and humidity based on gestational age and body weight. A water pillow was placed under the neck to prevent flexion, with body position changed every 2–3 h. All treatments, nursing care, procedures, and monitoring were performed within the incubator. A soft, 100% cotton infant blanket (150 cm × 75 cm) was rolled diagonally into a cylindrical shape, forming a nest with the occipital bone to lower limbs as the long axis and the width between shoulders as the short axis. The central section measured approximately 10 cm in height, while the seam area was about 5 cm high. Place an oval waterbed-style nest (made by filling 6–8 disposable gloves with hot water to 2/3 capacity, water temperature 38–40 °C) at the base. Place the nest in the incubator and preheat to 34–35 °C. After adjusting the incubator humidity, place the infant naked or in light clothing into the water-filled nest. Position the towel blanket's seam under the infant's head and shoulders, wrap the middle section around the lower limbs, and keep the limbs flexed near the body's midline. Elevate the bed board to 20°–30°. Monitor heart rate, respiration, and transcutaneous oxygen saturation continuously for 24 h using a neonatal multi-parameter monitor. (2) Continuous Improvement in breast milk feeding Quality: (1) Provide prenatal education on breast milk feeding benefits, milk collection, storage, and transportation, offering relevant learning materials to parents; (2) Initiate milk expression within 1 h postpartum (earlier is better), with frequent expression and breast massage; (3) Provide bedside, face-to-face education for mothers of newly admitted preterm infants to enhance understanding of breast milk feeding and improve skills; provide images and instruction manuals for various breast pump models; (4) Initiate pump-and-store within 2 h postpartum with 3-hourly pump reminders; (5) Address maternal concerns regarding diet, rest, sleep, and infant condition; (6) Record each pumping session's time and volume. Offer tailored interventions for mothers with low milk supply. Breast milk delivered to the NICU requires standardized management with immediate labeling to prevent contamination; (7) Refrigeration or Freezing: Deliver milk to the NICU within 1 day and store it in the refrigerator's fresh compartment. If delivery exceeds 1 day, store breast milk in the freezer compartment. Do not store breast milk in the freezer/door storage compartments. Fresh milk can be stored at room temperature (<25 °C) for 4 h, refrigerated (0 °C–4 °C) for 48–96 h, or frozen (–18 °C) for 3 months. Thawed milk or milk supplemented with fortifiers should be stored at 0 °C–4 °C for up to 24 h. When the infant's condition is stable and body weight reaches 2,000 g or more, discontinue ECG monitoring and incubator warming. Transfer the infant to a crib. The observation group should cease using the waterbed-style nest.

### 2.5 General demographic data

General demographic data for pediatric patients were collected through the medical record system, including gender (male/female), disease type (extremely low birth weight/very low birth weight), gestational age, birth weight, mode of delivery (cesarean section/vaginal delivery), conception method (natural conception/*in vitro* fertilization), maternal age, and paternal age.

### 2.6 Clinical course during hospitalization

Data collected and compared for all patients included: initiation time of pumped milk feeding, number of fasting episodes, duration of fasting, onset of lactation, average daily pumping frequency, time of first direct breast milk feeding, volume of direct breast milk consumed during hospitalization, initiation time of feeding, duration of microfeeding, duration of parenteral nutrition, and time to achieve full enteral feeding.

### 2.7 Quality of life

Collect and compare growth development indicators between groups: weight gain rate ([1,000 × ln(discharge weight/birth weight)]/(discharge age—age at birth weight recovery)), head circumference growth rate [(discharge head circumference—birth head circumference)/number of hospitalization weeks], length growth rate [(discharge length—birth length)/number of hospitalization weeks], time to birth weight recovery, and sleep duration.

### 2.8 Neurobehavioral development

Neurobehavioral developmental indicators were collected and compared before and after intervention. The Neonatal Assessment of Behavioral Neurodevelopment (NABA) (15) was used to evaluate pediatric patients’ neurobehavioral development, encompassing five dimensions: primitive reflexes (3 items), passive muscle tone (4 items), active muscle tone (4 items), general condition (3 items), and behavioral competence (6 items). Each item scored 0–2 points, yielding a total score of 0–40 points, where higher scores indicate better neurobehavioral development.

### 2.9 Serum markers

Serum levels of total protein (TP), albumin (ALB), and prealbumin (PA) were measured before and after intervention. Blood samples were collected at two standardized time points for all infants: (1) “before intervention,” defined as within 24 h prior to the initiation of the assigned management protocol; and (2) “after intervention,” defined as on the day of discharge from the NICU. Fasting venous blood (3 mL) was collected into heparinized tubes, centrifuged at 3,000 rpm for 10 min with a centrifuge radius of 8 cm. The supernatant serum was stored at −75 °C and analyzed using immunoturbidimetry.

### 2.10 Complications

Complications during hospitalization were collected and compared between the two groups, including feeding intolerance (defined as withdrawal of gastric tube before each feeding session, where gastric retention exceeded 50% of the feeding volume and was accompanied by abdominal distension and/or vomiting, bloody stools [occult blood], etc., thereby affecting continued enteral nutrition) (16), necrotizing enterocolitis (NEC) (diagnosed using the modified Bell staging system for NEC, with systemic manifestations such as unstable temperature, apnea, bradycardia, and lethargy, alongside gastrointestinal symptoms including abdominal distension, vomiting, and bloody stools; radiographic evidence of intestinal gas accumulation; stage II or higher diagnosed with these or more severe clinical and radiographic findings) (17), Bronchopulmonary dysplasia (BPD): Neonates requiring oxygen concentration >21% for >28 days. For those with gestational age <32 weeks, severity is classified based on corrected gestational age ≥36 weeks or discharge oxygen requirements: mild (no oxygen), moderate (oxygen concentration <30%), severe (oxygen concentration ≥30% or requiring mechanical ventilation). For gestational age ≥32 weeks, classified based on oxygen concentration at 56 days postnatal or at discharge: mild (no oxygen use), moderate (oxygen concentration <30%), severe (oxygen concentration ≥30%) (18); Late-onset sepsis (occurring >7 days postnatal, clinically characterized by signs of infection and toxemia; confirmed by positive blood culture; clinically diagnosed if blood culture is negative but ≥2 non-specific criteria are met [including elevated core temperature (>38.5 °C) or decreased core temperature (<36 °C), tachycardia or bradycardia, tachypnea, abnormal peripheral blood leukocyte count]) (19).

### 2.11 Statistical analysis

Data were analyzed using SPSS 25.0 statistical software. Count data were expressed as case numbers (percentages) and analyzed using the chi-square test or Fisher's exact test, as appropriate. Continuous variables meeting normal distribution were expressed as mean ± standard deviation (x̅ ± s) and analyzed using *t*-tests; those not meeting normal distribution were expressed as median and interquartile range [M(P25, P75)] and analyzed using Mann–Whitney *U* tests. A *P* value < 0.05 was considered statistically significant. To account for potential confounding variables, multivariate logistic regression analyses were performed for key binary outcomes (e.g., feeding intolerance, bronchopulmonary dysplasia). Variables included in the models were group assignment (observation vs. control), gestational age, birth weight, and gender.

## 3 Results

### 3.1 Comparison of general demographic characteristics between observation and control groups of extremely/very low birth weight infants

Comparisons of gender, disease type, gestational age, birth weight, mode of delivery, conception method, maternal age, and paternal age between the observation and control groups showed no statistically significant differences (*P* > 0.05), as shown in Table 1.

**Table 1:** Comparison of general demographic data of extremely/very low birth weight infants between the observation group and the control group.

Index		Observation Group (*n* = 53)	Control group (*n* = 50)	*x^2^/t*	*P*
Gender (*n*, %)	Male	27 (50.94)	30 (60.00)	0.854	0.355
	Female	26 (49.06)	20 (40.00)		
Disease type (*n*, %)	Very low birth weight (VLBW)	48 (90.57)	46 (92.00)	0.066	0.797
	Extremely low birth weight (ELBW)	5 (9.43)	4 (8.00)		
Gestational age (weeks, *x̅* ± s)		31.36 ± 2.29	31.41 ± 2.25	0.112	0.911
Birth weight (kg, *x̅* ± s)		1.16 ± 0.12	1.14 ± 0.11	0.880	0.381
Mode of delivery (*n*, %)	Cesarean section	32 (60.38)	30 (60.00)	0.002	0.969
	Natural delivery	21 (39.62)	20 (40.00)		
Conception method (*n*, %)	Natural conception	43 (81.13)	40 (80.00)	0.021	0.885
	*In vitro* fertilization	10 (18.87	10 (20.00)		
Mother's age (years, *x̅* ± s)		32.78 ± 3.32	32.62 ± 3.18	0.249	0.803
Father's age (years, *x̅* ± s)		33.01 ± 3.09	32.95 ± 3.10	0.098	0.922

### 3.2 Comparison of clinical outcomes during hospitalization between the observation group and control group of extremely low/very low birth weight infants

Comparisons between the observation and control groups for onset of lactation, average daily pumping frequency, hospital-acquired breast milk volume, initiation of feeding, duration of microfeeding, and time to achieve full enteral feeding showed no statistically significant differences (*P* > 0.05). However, the observation group demonstrated significantly earlier onset of pumping (15.43 ± 3.78 vs. 19.01 ± 3.57 h, *P* < 0.001), earlier time to first breast milk feeding (58.77 ± 9.33 vs. 64.33 ± 10.06 h, *P* = 0.004), fewer fasting episodes (1.68 ± 0.51 vs. 2.12 ± 0.33 times, *P* < 0.001), shorter fasting duration (3.22 ± 1.03 vs. 4.28 ± 1.34 days, *P* < 0.001), and shorter duration of parenteral nutrition (13.08 ± 2.37 vs. 16.56 ± 3.78 days, *P* < 0.001) compared to the control group, as shown in Table 2.

**Table 2:** Comparison of clinical conditions during hospitalization of extremely/very low birth weight infants in the observation group and the control group (*x̅* ± *s*).

Index	Observation Group (*n* = 53)	Control group (*n* = 50)	*t*	*P*
The time to start pumping milk (h)	15.43 ± 3.78	19.01 ± 3.57	4.935	<0.001
The start time of lactation (h)	26.34 ± 5.11	27.10 ± 4.89	0.770	0.443
The average number of milk pumps per day (time)	6.67 ± 1.92	6.94 ± 1.46	0.800	0.426
The time of the first breast milk feeding (h)	58.77 ± 9.33	64.33 ± 10.06	2.910	0.004
The amount of breast milk during hospitalization (L)	5.38 ± 1.43	5.71 ± 1.52	1.135	0.259
Number of fasts (time)	1.68 ± 0.51	2.12 ± 0.33	5.165	<0.001
Fasting days (d)	3.22 ± 1.03	4.28 ± 1.34	4.516	<0.001
Start feeding time (h)	35.69 ± 6.84	37.37 ± 6.92	1.239	0.218
Duration of microfeeding (d)	3.11 ± 0.89	3.33 ± 0.95	1.213	0.228
The duration of intravenous nutrition (d)	13.08 ± 2.37	16.56 ± 3.78	5.632	<0.001
The time to achieve total enteral feeding (d)	13.78 ± 3.16	14.88 ± 3.42	1.697	0.093

### 3.3 Comparison of quality of life between extremely low birth weight and very low birth weight infants in the observation and control groups

Body weight gain reflects recent nutritional status, head circumference correlates closely with brain development, height indicates long-term nutritional levels, time to regain birth weight assesses early growth stability, and adequate sleep is crucial for growth hormone secretion, directly influencing growth and development processes—all serving as vital indicators for early quality of life assessment. Sleep duration showed no statistically significant differences between the observation and control groups (*P* > 0.05). The observation group exhibited higher rates of weight gain [(16.56 ± 2.59 vs. 15.09 ± 2.95) g/(kg·d)], head circumference growth [(0.82 ± 0.09 vs. 0.73 ± 0.09) cm/week], and height growth rate [(1.11 ± 0.12 vs. 1.01 ± 0.15) cm/week] were higher than those in the control group. However, the time to regain birth weight [(8.90 ± 0.59 vs. 9.22 ± 0.95) days] was lower in the observation group than in the control group (*P* < 0.05), as shown in Table 3.

**Table 3:** Comparison of the quality of life of extremely/very low birth weight infants between the observation group and the control group (*x̅* ± *s*).

Index	Observation Group (*n* = 53)	Control group (*n* = 50)	*t*	*P*
Rate of increase in body mass[g/ (kg·d)]	16.56 ± 2.59	15.09 ± 2.95	2.691	0.008
The growth rate of head circumference (cm/weeks)	0.82 ± 0.09	0.73 ± 0.09	5.072	<0.001
Growth rate of body length (cm/weeks)	1.11 ± 0.12	1.01 ± 0.15	3.747	<0.001
The time to recover to birth weight (d)	8.90 ± 0.59	9.22 ± 0.95	2.066	0.041
Sleep duration (h)	14.38 ± 2.26	14.10 ± 2.02	0.645	0.520

### 3.4 Comparison of neurobehavioral development between observation and control groups of extremely low/very low birth weight infants

The NABA assessment evaluates neurological integrity and developmental status by measuring behavioral abilities, muscle tone, primitive reflexes, and other indicators, providing a basis for early intervention and personalized childcare. Prior to intervention, comparisons of primitive reflexes, passive muscle tone, active muscle tone, general condition, and behavioral ability scores between the observation and control groups showed no statistically significant differences (*P* > 0.05), as shown in Table 4.

**Table 4:** Comparison of neurobehavioral development of extremely/very low birth weight infants before intervention in the observation group and the control group (points, *x̅* ± *s*).

Index	Observation Group (*n* = 53)	Control group (*n* = 50)	*t*	*P*
Original reflection	2.15 ± 0.48	2.24 ± 0.49	0.941	0.349
Passive muscle tone	3.56 ± 0.52	3.45 ± 0.54	1.053	0.295
Active muscle tone	3.12 ± 0.61	3.02 ± 0.59	0.845	0.400
General state	2.23 ± 0.52	2.18 ± 0.56	0.470	0.639
Capacity for conduct	8.05 ± 0.88	8.14 ± 0.91	0.510	0.611

**Figure 1:**
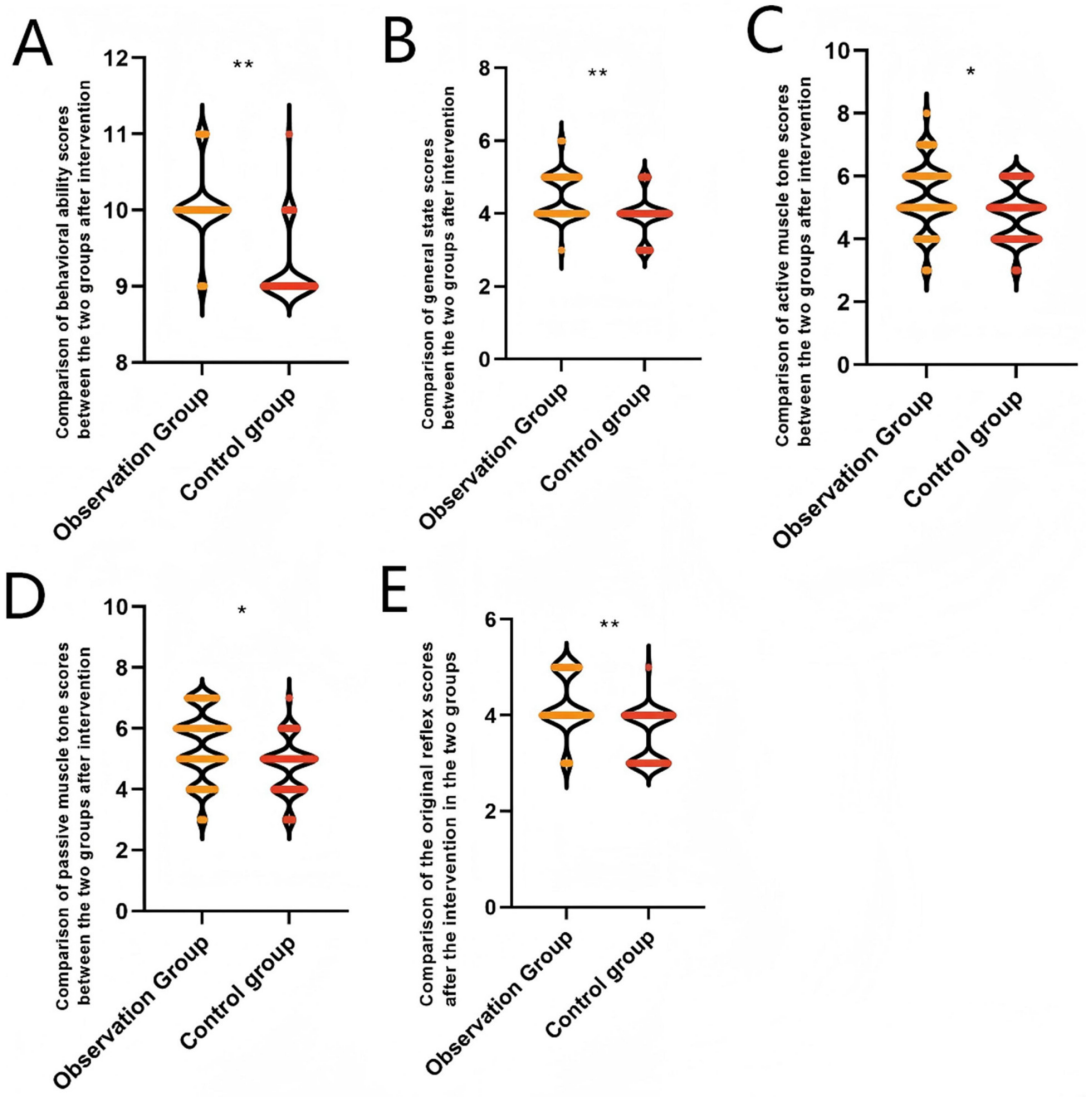
Comparison of neurobehavioral development of extremely/very low birth weight infants after intervention between the observation group and the control group. **(A)** Comparison of behavioral ability scores between the two groups after intervention. **(B)** Comparison of general state scores between the two groups after intervention. **(C)** Comparison of active muscle tone scores between the two groups after intervention. **(D)** Comparison of passive muscle tone scores between the two groups after intervention. **(E)** Comparison of the original reflex scores. *P < 0.05, **P < 0.001.

After intervention, the observation group showed significant improvements in primitive reflexes (4.23 ± 0.61 vs. 3.59 ± 0.55 points), passive muscle tone (5.45 ± 1.11 vs. 4.76 ± 0.89 points), active muscle tone (5.36 ± 1.08 vs. 4.81 ± 0.86 points), general condition (4.45 ± 0.62 vs. 3.94 ± 0.57 points), and behavioral capacity (10.06 ± 0.51 vs. 9.21 ± 0.48 points) were significantly higher than those in the control group (*P* < 0.05). indicating that the waterbed nest combined with a continuous breast milk feeding quality improvement program effectively enhances behavioral and neurodevelopmental functions in pediatric patients, as shown in Figure 1.

### 3.5 Comparison of serum markers between observation and control groups in extremely low/very low birth weight infants

Total protein (TP) and albumin (ALB) reflect hepatic synthetic function and nutritional reserves, while protein turnover (PA) rapidly indicates changes in body protein metabolism. All are crucial indicators for evaluating parenteral nutrition efficacy and protein nutritional status. Before intervention, comparisons of TP, ALB, and PA levels between the observation and control groups showed no statistically significant differences (*P* > 0.05), as shown in Table 5.

**Table 5:** Comparison of serum indicators of extremely/very low birth weight infants in the observation group and the control group before intervention.

Index	Observation Group (*n* = 53)	Control group (*n* = 50)	*t*	*P*
TP (g/L)	52.38 ± 4.13	53.05 ± 4.15	0.821	0.414
ALB (g/L)	33.56 ± 4.11	34.15 ± 4.16	0.724	0.471
PA (mg/L)	83.45 ± 7.42	83.89 ± 7.19	0.305	0.761

**Figure 2:**
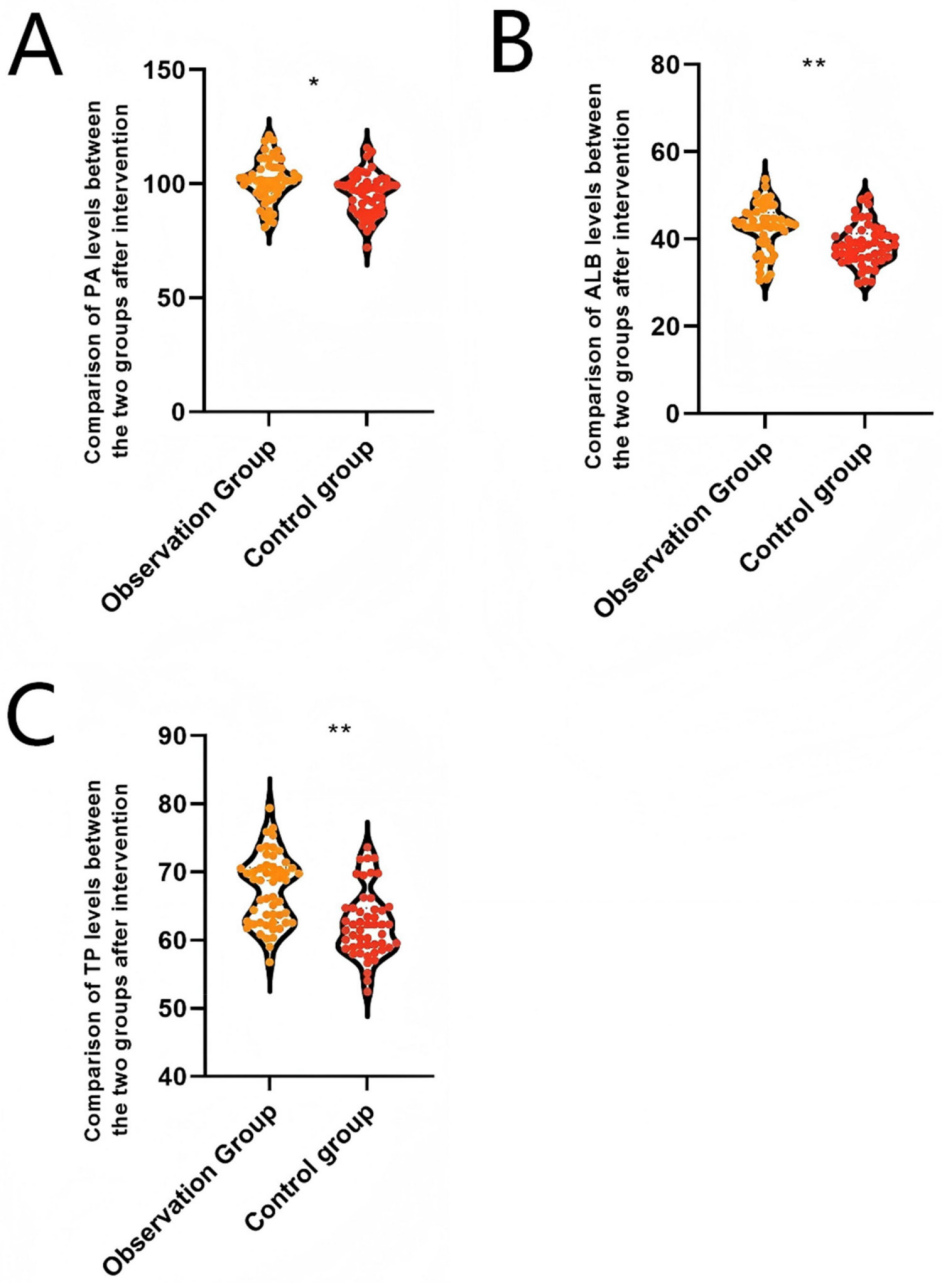
Comparison of serum indicators of extremely/very low birth weight infants in the observation group and the control group after intervention. **(A)** Comparison of PA levels between the two groups after intervention. **(B)** Comparison of ALB levels between the two groups after intervention. **(C)** Comparison of TP levels between the two groups after intervention. TP denotes total protein, ALB denotes albumin, PA denotes prealbumin; *P < 0.05, **P < 0.001.

Following intervention, patients in the observation group exhibited significantly higher levels of TP [(67.42 ± 5.11 vs. 62.33 ± 4.86) g/L], ALB [(42.66 ± 5.39 vs. 38.69 ± 4.68) g/L], and PA [(101.35 ± 9.34 vs. 95.41 ± 9.11) mg/L] levels were significantly higher than those in the control group (*P* < 0.05), indicating that the waterbed-style bird's nest combined with a continuous improvement program for breast milk feeding quality effectively enhanced the nutritional status of pediatric patients, as shown in Figure 2.

### 3.6 Comparison of complications in extremely low birth weight and very low birth weight infants between observation and control groups

Complications serve as an indicator for assessing the safety of management protocols; a lower overall complication rate suggests greater safety of the management approach. The incidence of necrotizing enterocolitis and late-onset sepsis showed no statistically significant difference between the two groups (*P* > 0.05). However, the incidence of feeding intolerance (20.75% vs. 46.00%), bronchopulmonary dysplasia (5.66% vs. 20.00%) were significantly lower in the observation group than in the control group (*P* < 0.05). This suggests that the waterbed nest combined with a continuous improvement program for breast milk feeding quality can effectively reduce feeding intolerance and bronchopulmonary dysplasia in infants, as shown in Table 6.

**Table 6:** Comparison of complications in extremely/very low birth weight infants between the observation group and the control group (*n*, %).

Index	Observation Group (*n* = 53)	Control group (*n* = 50)	*x* ^2^	*P*
Feeding intolerance	11 (20.75)	23 (46.00)	7.415	0.006
Necrotizing enterocolitis	3 (5.66)	5 (10.00)	0.676	0.411
Bronchopulmonary dysplasia (Overall)	3 (5.66)	10 (20.00)	4.797	0.029
Stratified by Birth Weight				
VLBW (birth weight 1,000–1,499 g)	2/48 (4.17)	8/46 (17.39)	4.167	0.041
ELBW (birth weight <1,000 g)	1/5 (20.00)	2/4 (50.00)	0.900	0.343
Stratified by Ventilatory Support*				
Received MV/NCPAP	3/45 (6.67)	10/44 (22.73)	4.585	0.032
No MV/NCPAP	0/8 (0.00)	0/6 (0.00)	–	–
Late-onset sepsis	8 (15.09)	10 (20.00)	0.429	0.512

* MV, mechanical ventilation; NCPAP, nasal continuous positive airway pressure.

## 4 Discussion

With changes in China's family planning policy, the increasing number of older pregnant women and newborns has led to a phased rise in preterm birth rates. Preterm birth remains a primary cause of mortality among newborns and children under five, posing a significant global public health challenge (20, 21). Sharif S et al. (22) indicate that the incidence of extremely low birth weight (ELBW) infants reaches 0.1%. Although this rate is relatively low, ELBW infants face higher risks of complications such as infections, visual impairments, and intellectual disabilities compared to normal-weight infants. Therefore, implementing scientifically sound management strategies is crucial for reducing complications and promoting normal development in these infants. Conventional nursing interventions provide adequate care through fundamental measures like respiratory management, nutritional support, anemia management, and complication prevention. However, these approaches often lack sufficient humanistic care, focusing solely on immediate disease resolution while neglecting long-term growth and development monitoring, resulting in suboptimal outcomes (23). In this study, the observation group demonstrated higher rates of weight gain, head circumference growth, and length growth compared to the control group. However, the observation group had earlier initiation of pumped milk feeding, earlier first direct breast milk feeding, fewer fasting episodes, shorter fasting duration, shorter duration of parenteral nutrition, and faster recovery to birth weight than the control group. Post-intervention, the observation group demonstrated significantly higher scores in primitive reflexes, passive muscle tone, active muscle tone, general condition, behavioral capacity, total protein (TP), albumin (ALB), and phosphorus (PA) compared to the control group (*P* < 0.05). This indicates that implementing a waterbed-style nest combined with continuous quality improvement management for breast milk feeding in extremely/very low birth weight infants holds significant clinical value.

Multiple studies indicate that breast milk is the preferred enteral nutrition for newborns. For preterm and critically ill infants in the NICU, breast milk feeding not only promotes growth, maturation, and protective effects but also reduces intestinal feeding intolerance. In recent years, quality improvement has been widely applied in clinical practice. Through targeted, comprehensive interventions tailored to specific clinical scenarios, it avoids subjectivity and limitations, providing reliable approaches for clinical quality enhancement. Its practical application in NICUs has demonstrated its critical importance for neonatal health (24–26). In this study, the observation group received a combined intervention of waterbed-style nesting and a breast milk feeding quality improvement program. The synergistic effect of this combined approach significantly reduced the time to initiate pumping, duration of intravenous nutrition, and number of fasting episodes. The breast milk feeding quality improvement component substantially promoted milk secretion, addressing delayed lactation onset due to prolonged pumping initiation. It also partially resolved insufficient milk supply caused by delayed pumping start times, reduced complications associated with intravenous nutrition, and improved the quality of life for preterm infants. Patra K et al. (27) demonstrated that gut bacteria can be transported from the intestinal lumen to the mammary gland via an endogenous pathway mediated by monocytes. Thus, beyond direct fecal-oral transmission, gut commensal bacteria may also undergo vertical transmission through the gut-mammary pathway, profoundly influencing the nutritional status of infants. In this study, the observation group implemented breast milk quality improvement measures. The breast milk contained live bacteria capable of colonizing the neonatal gut, with dominant bacterial groups including staphylococci, streptococci, bifidobacteria, and lactobacilli. Breast milk samples obtained via pump exhibited higher total bacterial counts and a greater proportion of enterobacteria. These components stimulate oropharyngeal lymphoid tissue, activate T cells, and influence oropharyngeal microbial colonization, thereby conferring immunity. Simultaneously, oral interventions promote gastrointestinal fluid secretion and intestinal motility, improving feeding and nutritional status while enhancing the infant's quality of life.

A primary limitation of this study is the bundled nature of the intervention. Because the waterbed-style nest and the breastfeeding quality improvement program were implemented simultaneously, it is not possible to disentangle their individual effects or determine the precise causal pathway leading to the observed outcomes. For example, the reduction in bronchopulmonary dysplasia could be attributable to the improved nutritional and immunological benefits from optimized breast milk feeding, the reduced physiological stress from the supportive nest, or, more likely, a combination of both. Therefore, while the combined protocol demonstrates significant clinical value, we cannot attribute the improvements to any single component. Future studies employing a factorial design would be necessary to isolate and quantify the independent contribution of each intervention.

The integration of the waterbed-style nest with this nutritional support created a comprehensive care environment. Specifically, the waterbed-style nest provided a more maternal-like environment for newborns. When the infant moves limbs or caregivers perform procedures, the water in the base bed flows rhythmically, producing sounds similar to amniotic fluid in the womb. This satisfies the newborn's need for security and emotional connection while promoting rhythmicity and stimulating vestibular movement. For newborns, this environment is safer and more comfortable, thereby benefiting their growth and development (28, 29). The waterbed-style nest management restricted the infants’ range of motion, maintaining their head and neck in a normal flexed position. This facilitated the normal development of the mandibular muscle groups, enhanced muscle strength, improved the coordination of sucking, swallowing, and breathing, and strengthened their sucking, swallowing, and respiratory capabilities. Touching the cheeks with both hands increased head-face interaction, facilitating finger sucking to further enhance sucking ability, boost security, and increase spontaneous milk intake; Maintaining a posture resembling the intrauterine environment provides a sense of boundaries and security, alleviating inner fear and tension. This improves digestive function, reduces gastric tube reflux, enables earlier removal of the gastric tube, shortens the duration of total parenteral nutrition, ventilator use, and hospitalization, decreases the incidence of feeding intolerance, and enhances nutritional status. As referenced in Huang Z et al.'s (30) study, implementing a waterbed-style nest with shoulder flexion and hands touching the cheeks stimulates neuronal development and synaptic connections, promoting tactile development and improving behavioral neurodevelopment. Furthermore, the physiological curvature of the hips and lower limbs reduces patient positional shifts, protects organs like the lungs and stomach, facilitates respiratory and digestive system development, lowers the incidence of complications such as iatrogenic non-suture closure cranial deformities caused by poor posture, and enhances lower limb muscle strength, further promoting behavioral and neurological development in infants. The combination of these interventions likely worked synergistically: the secure positioning reduced energy expenditure and stress, allowing the infants to better utilize the nutrients and immunological components provided by the optimized breast milk feeding protocols, thereby improving weight gain, nutritional status, and neurodevelopmental outcomes.

Research both domestically and internationally indicates that breast milk feeding can reduce the incidence of feeding intolerance, sepsis, necrotizing enterocolitis, and bronchopulmonary dysplasia, prevent retinopathy of prematurity, and promote neurological development (31). Data from this study indicate that the incidence rates of feeding intolerance and bronchopulmonary dysplasia (BPD) were significantly lower in the intervention group than in the control group (*P* < 0.05). To further explore this finding, we performed a stratified analysis (Table 6). The reduction in BPD remained statistically significant in the VLBW subgroup and in infants who received any form of positive pressure ventilation, suggesting the intervention's potential benefit extends across these clinically relevant categories. However, the lack of significance in the small ELBW subgroup (*n* = 9) is likely due to insufficient statistical power. These stratified results, while suggestive, should be interpreted with caution and require validation in larger, adequately powered studies. This suggests that the waterbed-style bird's nest combined with continuous improvement in breast milk feeding quality can reduce the incidence of feeding intolerance and bronchopulmonary dysplasia, consistent with domestic and international research. This effect stems from the waterbed-style bird's nest simulating the intrauterine environment by maintaining neutral temperature and minimizing heat loss. Elevating the infant's head and neck by 20°–30° with slight neck extension straightens the airway, alleviates esophageal compression, and ensures effective respiration—providing a secure foundation for breast milk feeding. Breast milk, rich in immunologically active substances, enhances intestinal immunity, reduces infection and inflammation-induced lung damage, and together with the waterbed nest, blocks the progression of bronchopulmonary dysplasia. This combination also optimizes nutrient absorption and lowers the risk of feeding intolerance. In this study, the incidence of necrotizing enterocolitis and late-onset sepsis showed no statistically significant difference between the two groups. This may be attributed to factors such as the small sample size of extremely preterm/very low birth weight infants and the relatively low number of complications in both groups. Therefore, these findings require further validation through large-scale prospective studies. However, this study has limitations: it is a single-center, small-sample retrospective design, which may yield unstable or non-representative results due to sample size and selection bias. Additionally, the lack of clinical follow-up prevents a comprehensive assessment of the long-term advantages and limitations of the waterbed nest combined with continuous improvement in breast milk feeding quality. Long-term clinical observation and follow-up are necessary to evaluate its sustained efficacy. Consequently, future large-scale, multicenter, prospective studies are needed to avoid bias from small sample sizes. Long-term follow-up of subjects is essential to evaluate long-term efficacy and safety, conduct in-depth analysis of the sustained benefits of waterbed-style bird's nest combined with continuous improvement in breast milk feeding quality, and provide theoretical support for clinical practice.

## 5 Conclusions

In this retrospective cohort study, the use of waterbed-style nests combined with continuous improvement in breast milk feeding quality for extremely low birth weight infants demonstrated significant clinical value. This approach was associated with improved short-term physical growth and neurobehavioral function, demonstrating high clinical efficacy and safety during the initial hospitalization. It also reduces complications such as feeding intolerance and bronchopulmonary dysplasia. However, due to the absence of long-term follow-up data, we cannot conclude that these early benefits translate into an enhanced long-term quality of life. Future research with longitudinal follow-up and standardized neurodevelopmental assessments is warranted to evaluate the sustained impact of this intervention.

## Data Availability

The original contributions presented in the study are included in the article/Supplementary Material, further inquiries can be directed to the corresponding author.
